# Infection of Human Endothelial Cells by Japanese Encephalitis Virus: Increased Expression and Release of Soluble HLA-E

**DOI:** 10.1371/journal.pone.0079197

**Published:** 2013-11-13

**Authors:** Shwetank, Onkar S. Date, Kwang S. Kim, Ramanathapuram Manjunath

**Affiliations:** 1 Department of Biochemistry, Indian Institute of Science, Bangalore, Karnataka, India; 2 Department of Pediatric Infectious Diseases, John Hopkins University School of Medicine, Baltimore, Maryland, United States of America; Utah State University, United States of America

## Abstract

Japanese encephalitis virus (JEV) is a single stranded RNA virus that infects the central nervous system leading to acute encephalitis in children. Alterations in brain endothelial cells have been shown to precede the entry of this flavivirus into the brain, but infection of endothelial cells by JEV and their consequences are still unclear. Productive JEV infection was established in human endothelial cells leading to IFN-β and TNF-α production. The MHC genes for HLA-A, -B, -C and HLA-E antigens were upregulated in human brain microvascular endothelial cells, the endothelial-like cell line, ECV 304 and human foreskin fibroblasts upon JEV infection. We also report the release/shedding of soluble HLA-E (sHLA-E) from JEV infected human endothelial cells for the first time. This shedding of sHLA-E was blocked by an inhibitor of matrix metalloproteinases (MMP). In addition, MMP-9, a known mediator of HLA solubilisation was upregulated by JEV. In contrast, human fibroblasts showed only upregulation of cell-surface HLA-E. Addition of UV inactivated JEV-infected cell culture supernatants stimulated shedding of sHLA-E from uninfected ECV cells indicating a role for soluble factors/cytokines in the shedding process. Antibody mediated neutralization of TNF-α as well as IFNAR receptor together not only resulted in inhibition of sHLA-E shedding from uninfected cells, it also inhibited HLA-E and MMP-9 gene expression in JEV-infected cells. Shedding of sHLA-E was also observed with purified TNF-α and IFN-β as well as the dsRNA analog, poly (I:C). Both IFN-β and TNF-α further potentiated the shedding when added together. The role of soluble MHC antigens in JEV infection is hitherto unknown and therefore needs further investigation.

## Introduction

Viral encephalitis caused by Japanese encephalitis virus (JEV) is a mosquito-borne disease that is prevalent in different parts of India and South East Asia [1], [2]. JEV is a positive sense single stranded RNA virus that belongs to the *Flavivirus* genus of the family *Flaviviridae* [3]. This neurotropic virus as well as its ability to cause encephalitis has been well studied with respect to its structural, pathological, immunological and epidemiological aspects [4], [5], [6], [7], [8]. After entry into the host following a mosquito bite, JEV infection leads to acute peripheral neutrophil leucocytosis in the brain and elevated levels of type I interferon, macrophage-derived chemotactic factor, RANTES, TNF-α and IL-8 in the serum and cerebrospinal fluid [9]. Recently, limited amplification of JEV was shown in rat endothelial cells [10]. Flaviviruses such as West Nile (WNV) and dengue viruses [11], [12], [13] have been shown to infect endothelial cells and several neurotropic viruses that infect brain parenchymal cells also infect endothelial cells [14]. These observations lend support to the speculations that virion budding on the parenchymal side of infected endothelial cells [15], transcytosis across cerebral capillary endothelial cells and infiltration of infected leukocytes across a compromised blood brain barrier (BBB) could be the mechanisms of CNS invasion [16], [17].

Flaviviruses are known to upregulate MHC expression in mouse models and in human cells [18], [19]. Flavivirus-induced cell surface expression of classical MHC-I is thought to confer protection to infected cells against lysis by Natural Killer (NK) cells. It is hence regarded as an immunoevasive strategy developed by flaviviruses against host mediated innate immune responses. Cell surface expression of the non-classical MHC-I molecule, HLA-E is also known to lead to NK inhibition by binding to CD94/NKG2A receptors on NK cells [20]. However, it is not known whether JEV can infect human endothelial cells or that this infection could result in upregulation of HLA genes. Hence it was of interest to study the effects of JEV infection and its ability to induce HLA-A, -B, -C and HLA-E molecules in human endothelial cells. HLA-A, -B and –C are members of the classical human MHC-I complex, while HLA-E is a non-classical human MHC-I molecule.

Here we present the evidence that immortalized human brain microvascular endothelial cells (HBMEC) and the endothelial-like cell line, ECV304 (ECV) can be productively infected with JEV leading to the production of IFN-β and TNF-α. Both these cell lines were selected since they are used in several *in vitro* model studies as an endothelial component of the human BBB [21], [22]. Human foreskin fibroblasts (HFF) were also included in our studies for comparison since fibroblasts have been used both in human and mouse models to study the effects of flavivirus infection *in vitro* [23], [24], [25], [26], [27]. Infection of human fibroblasts with WNV, also a flavivirus leads to limited replication and increased cell surface expression of MHC molecules [19].

JEV infection induced the expression of HLA-A, -B and HLA-E genes in all these cell types. However, infection of endothelial cells led to shedding of HLA-E molecules, but in contrast, JEV infection of HFF cells resulted in only upregulation of HLA-E expression on the cell surface. More importantly, JEV induced shedding of soluble HLA-E (sHLA-E) from infected HBMEC and ECV cells could be partially blocked by matrix metalloproteinase (MMP) inhibition. Further, inhibition studies showed that both anti-TNFα, and IFNAR antibodies were required to block sHLA-E release from infected and uninfected ECV cells.

## Results

### Induction of HLA Class I by JEV Infection

**Figure 1:**
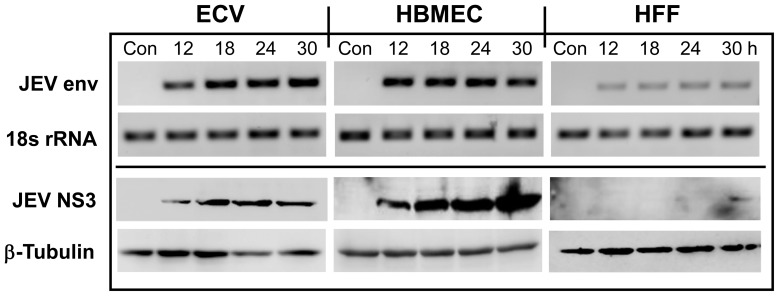
JEV infection of human endothelial cells. Top Panel: Total RNA from ECV, HBMEC and HFF cells was subjected to semiquantitative RT-PCR analysis for JEV envelope and control 18s rRNA. Bottom Panel: Cell lysates (100 µg protein) from ECV, HBMEC and HFF were subjected to Western blotting using rabbit anti JEV-NS3 and control goat anti-β-tubulin antibodies. Both panels show uninfected cells (Con) and cells infected for 12 h, 18 h, 24 h or 30 h.

HBMEC, ECV and HFF cells were first tested for their ability to support JEV infection. Both ECV and HBMECs supported JEV infection and replication as judged by the RT-PCR amplification of JEV envelope RNA (Fig. 1, top panel), the presence of the NS3 nonstructural protein of JEV (Fig. 1, bottom panel) and viral titers produced at different times of infection (Table. S1). In contrast, the ability of HFF cells to support productive JEV infection was found to be rather limited, confirming earlier reports with WNV, a related flavivirus [19]. Although signals for viral envelope RNA were present, no synthesis of the JEV-NS3 protein (Fig. 1, bottom panel) and neither viral PFU (Table. S1) or viral cytopathic effects were detectable. This suggested that HFF cells could be undergoing abortive infection. Abortive infection of cells resulting in the synthesis of some but not all viral proteins has been demonstrated for other viruses [28], [29].

The ability of JEV infected ECV, HBMEC and HFF cells to upregulate HLA class I transcripts was then examined since JEV induces the cell surface expression of MHC-I on mouse fibroblasts [30]. Real time RT-PCR analysis showed that JEV infection results in increased transcription of HLA-A, -B, -C and –E genes. Maximum fold changes of 11.9, 10.9 and 5 were observed for the transcription of HLA-B in ECV, HBMEC and HFF respectively at 30 h after infection. Among the three cell lines, induction of the HLA-E gene in HFF was maximal (3.2 fold) at 30 h after infection (Table. 1).

**Table 1:** Real Time PCR analysis of HLA gene transcription.

HLA	Time of infection	Cell Line^@^		
	(Hours)	HBMEC	ECV	HFF
**HLA-A**				
	12	0.9±0.06	0.7±0.02	1.2±0.01
	18	2.2±0.09*	1.1±0.22	1.5±0.03
	24	3.4±0.10*	1.9±0.10	2.0±0.02*
	30	3.5±0.12*	3.2±0.10*	2.6±0.01*
**HLA-B**				
	12	0.5±0.08	1.2±0.30	1.3±0.11
	18	2.5±0.09*	1.6±0.20	2.3±0.10*
	24	8.0±0.05*	6.5±0.12*	3.4±0.10*
	30	10.9±0.15*	11.9±0.10*	5.0±0.01*
**HLA-C**				
	12	1.0±0.03	1.9±0.13	1.5±0.02
	18	1.1±0.03	1.2±0.20	2.0±0.10*
	24	1.3±0.10	0.8±0.02	2.6±0.01*
	30	1.3±0.11	0.6±0.10	3.8±0.01*
**HLA-E**				
	12	1.0±0.08	1.7±0.24	2.1±0.20#
	18	1.3±0.12	1.3±0.11	2.8±0.20*
	24	1.5±0.09	1.9±0.12	3.3±0.10*
	30	1.8±0.11	2.5±0.30*	3.2±0.10*

@ Data represent Mean fold change relative to uninfected.

controls ± SEM of triplicate assays.

* P value <0.001,

# P value <0.01.

**Figure 2:**
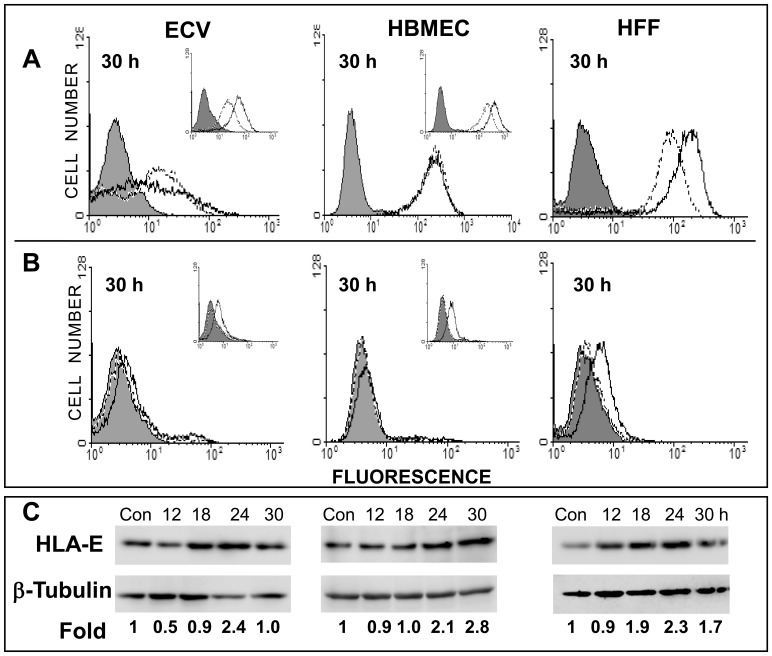
JEV infection and expression of HLA antigens. As labeled, ECV, HBMEC and HFF were stained for the cell surface expression of total HLA class I (Panel A) and HLA-E (Panel B) at 30 h after JEV infection. Alterations in cell surface expression of total HLA class I and HLA-E were not observed when tested at earlier times of infection and have not been shown. Filled histograms represent cells stained with control antibody while dotted lines represent antigen-specific staining with uninfected cells and solid lines represent antigen-specific staining with JEV-infected or IFN-γ treated cells. Insets in both panels represent cells treated for 24 h with 500 IU/ml IFN-γ. Panel C represents Western blotting analysis of cell lysates from uninfected cells (Con) and cells infected for the labeled times. Numbers at the bottom indicate the fold change in banding intensities of HLA-E after normalization to β-tubulin.

Next, we analysed the cell surface expression of these HLA molecules. Cell surface expression of total HLA antigens (-A, -B and -C), was evaluated by the pan HLA-specific monoclonal antibody, W6/32. Despite upregulation of HLA-A and -B at the transcript level, ECV and HBMEC cells failed to show increased cell surface expression of total HLA antigen in response to JEV infection at 30 h p.i. (Fig. 2A). Cell surface expression of HLA-E also remained unchanged in HBMECs while the increase obtained in ECV cells was only modest. Alterations in cell surface of total HLA class I and HLA-E were also not observed when tested at earlier times (unpublished data). In contrast, JEV-infected HFF cells showed a perceptible increase in cell surface expression of both total HLA (-A, -B and -C) and HLA-E (Fig. 2A and 2B) despite its limited ability to support infection. Treatment with IFN-γ, a known inducer of HLA-E [31] increased the expression of total HLA class I (Fig. 2A, Insets) as well as HLA-E (Fig. 2B, Insets) on the cell surface of ECV and HBMEC, indicating that these cells were not defective in HLA class I induction. Western blotting showed that total intracellular HLA-E protein within infected ECV, HBMEC and HFF cells increased, but only by about 2 fold at 24 h after JEV infection (Fig. 2C). In contrast to the above cell lines, increases in total HLA-E protein were easily detectable in control studies using other JEV-infected cell lines such as AV-3, FL and WISH amniotic epithelial cells (Fig. S1A). These results ascertained that the JEV-induced induction of HLA-E protein could be observed in other cell types in addition to ECV and HBMECs.

### Release of Soluble HLA-E upon JEV Infection

The above data indicated that the JEV-induced increases in HLA gene transcription were not translated into changes in protein levels at the endothelial cell surface. Studies on human endothelial cells and other melanomas report that HLA-E can be shed upon treatment with IFN-γ, IL-1β or TNF-α [32], [33]. Hence we checked whether HLA-E molecules were released into the infected cell culture supernatants upon JEV infection.

**Figure 3:**
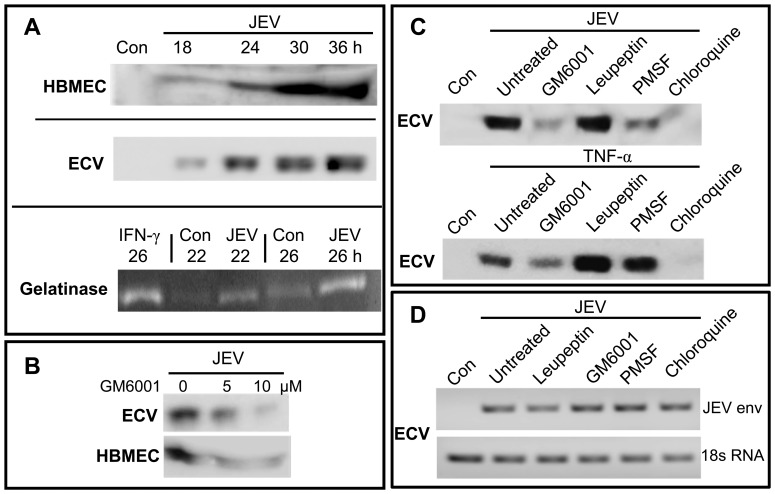
Shedding of sHLA-E into JEV-infected culture supernatants. Panel A: Western blot for sHLA-E in cell-culture supernatants from uninfected (Con) and infected HBMEC and ECV at various times p.i. as labeled. ‘Gelatinase’ represents enzyme activity in normal (Con) ECV cells that were either treated with IFN-γ or infected with JEV for the indicated times. Panel B: Cell-culture supernatants were collected from ECV or HBMEC cells that were infected for 24 h with JEV and cultured in the presence of 0 µM, 5 µM and 10 µM GM6001. Panel C: Cell-culture supernatants were collected from uninfected (Con) and 24 h JEV-infected (Top) or TNF-α treated (Bottom) ECV cells that were left untreated or treated with 10 µM GM6001, 10 µM leupeptin, 1 mM PMSF, and 100 µM chloroquine. Panel D: Total RNA was isolated from uninfected (Con) and 24 h JEV-infected ECV cells that were left untreated or treated with 10 µM GM6001, 10 µM leupeptin, 1 mM PMSF, and 100 µM chloroquine. RT-PCR was performed for the JEV envelope and 18s RNA controls. All inhibitors used in panels B, C and D were present only during the last 8 h of the 24 h-culture.

As shown in Fig. 3A, JEV infection results in a time-dependent release of sHLA-E in HBMEC and ECV (top and middle panels) beginning at 18 h p.i. when cell viability was greater than 90%. We, then assayed the gelatinase activity of MMPs in JEV-infected ECV culture supernatants since MMPs have been shown to release HLA molecules from the cell surface [34]. While a low basal level activity was observed in control ECV supernatants, (Fig. 3A, bottom panel), the activity increased further at 22 h and 26 h p.i. concomitant to the shedding of HLA-E from infected ECV cells.

MMP mediated proteolysis of the 42 kDa membrane-bound HLA-E results in the release of a 37 kDa soluble form [33] (Fig. S1B). Hence, we tested the effect of protease inhibitors on JEV-induced shedding of sHLA-E. The shedding of sHLA-E from ECV (Fig. 3B, top panel) and HBMECs (Fig. 3B, bottom panel) was inhibited by GM6001, a broad spectrum MMP inhibitor [32]. However, leupeptin, a thiol proteinase inhibitor failed to inhibit the release of sHLA-E and PMSF, a serine proteinase inhibitor partially blocked it (Fig. 3C, top panel). Since IFN-γ is reported to induce the shedding of sHLA-E from melanoma cells in a MMP dependent manner [33], we determined if TNF-α could also induce sHLA-E shedding from ECV cells and if it could be inhibited by the above inhibitors. As shown in Fig. 3C, (bottom panel), TNF-α treatment also led to sHLA-E shedding that was inhibited by GM6001 but not by leupeptin as in the case of JEV infection. However, PMSF was unable to inhibit the effect of TNF-α suggesting that the PMSF mediated inhibition of sHLA-E shedding from infected ECV cells was possibly due to its inhibition of JEV-NS3 protease mediated virus maturation (Table. S2). Chloroquine, a lysozomal inhibitor blocked both JEV-induced and TNF-α induced (Fig. 3C) shedding of sHLA-E. This inhibitory effect of choloroquine on sHLA-E shedding was possibly due to a block in HLA-E recycling to the membrane as hypothesized by an earlier study [32]. All inhibitors were used only during last 8 h of assay and GM6001 did not alter virus production, recovery of viable cells or viral RNA (Table. S2 and Fig. 3D).

In addition to HLA-E, Western blot analysis using native PAGE gels showed that HLA-A, and -B were also shed from 24 h JEV infected ECV but not HFF cells (Fig. S2). Mobilities of the released molecules on native PAGE gels were confirmed by IFN-γ induced cell-culture supernatant controls (Fig. S2, Lanes 9 and 10). However, we focused our attention on HLA-E due to the paucity of a pan HLA antibody that could detect HLA-A, -B and –C on denaturing PAGE, as well as the possibility that soluble classical HLA antigens could comprise several different forms [35] that could make results difficult to interpret in different cell lines.

### JEV Induces MMP-9 Gene Expression in ECV Cells

Upregulation of MMPs by mouse hepatitis virus (JHMV) in the CNS [36] and by JEV in rat brain astrocytes has been reported [37]. The shedding of sHLA-E from endothelial cells activated by TNF-α, IL-1 and IFN-γ in vascular diseases has also been reported to be MMP-9 dependent [32]. Our data obtained with GM6001 and gelatinase prompted us to quantify the gene expression of MMP-9 in JEV-infected cells. MMP-9 gene expression was induced 38 fold in ECV and 28 fold in HBMEC at 30 h after infection (Table. 2). ECV cells failed to show MMP-2 gene expression either before or after JEV infection and HFF cells did not shed sHLA-E despite constitutive MMP-2 gene expression (unpublished data). Together, our data confirm earlier reports [33] that suggested the participation of MMPs in the shedding of sHLA-E from melanoma cells.

**Table 2:** Real Time PCR analysis of MMP-9 gene transcription.

Time of infection	Cell Line^@,^ §		
(Hours)	HBMEC	ECV	HFF
6	nd†	6.5±0.22*	1.8±0.20
12	3.5±0.20	11.7±0.04*	1.4±0.13
18	4.9±0.50#	12.3±0.13*	0.8±0.10
24	9.5±0.80*	17.2±0.10*	1.0±0.10
30	27.7±0.20*	38.1±0.10*	2.6±0.20*

@ Data represent Mean fold change relative to untreated.

controls ± SEM of triplicate assays.

§ ECV cells that were mock infected for 18, 24 and 30 h.

did not result in significant changes in gene expression.

* P value <0.001,

# P value <0.01.

† nd: not determined.

### Shedding of sHLA-E by Activating Agents

**Figure 4:**
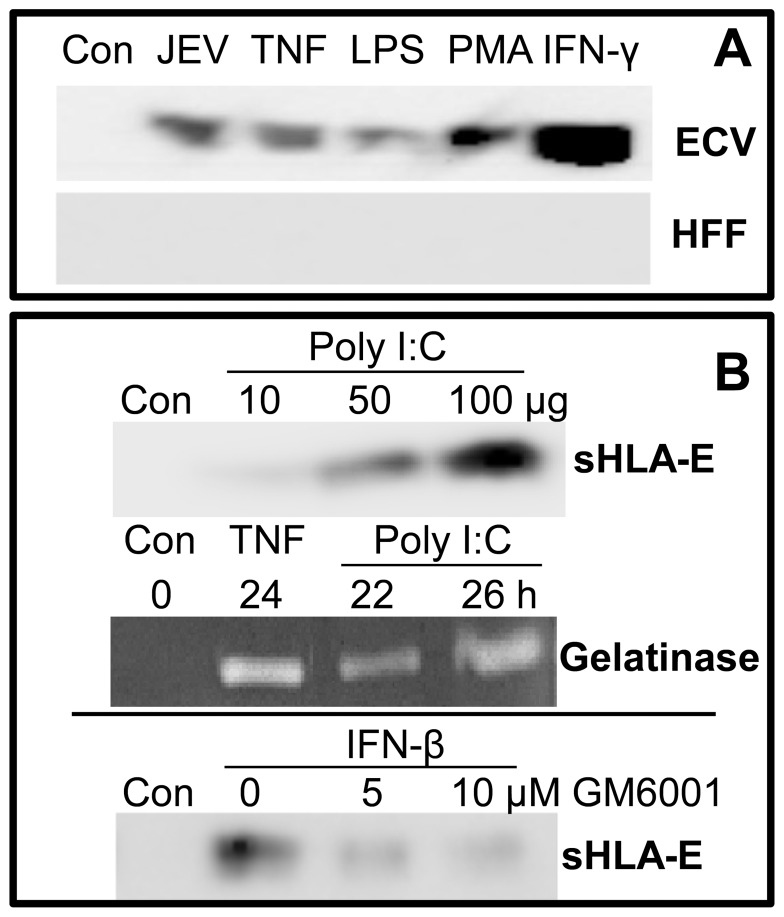
Shedding of HLA-E by activating agents. Panel A: Shedding of sHLA-E from ECV and HFF cells is shown for cells that were either uninfected (Con), 24 h JEV-infected or treated with TNF-α, LPS, or IFN-γ as labeled. Panel B: Top panel shows shedding of sHLA-E from normal cells (Con) and cells that were treated for 24 h with different amounts of poly I:C. Middle panel shows gelatinase activity in normal cells (Con) and cells that were treated with TNF-α or poly I:C (100 µg) for the labeled times. Bottom panel represents sHLA-E shedding from normal cells (Con) and cells that were treated with IFN-β for 24 h, in the absence or presence of GM6001 during the last 8 h of culture.

Because HFF responded differently to JEV infection and failed to show shedding of sHLA-E (Fig. 4A), we asked whether different activating agents could induce HFF cells to shed sHLA-E. We used TNF-α and IFN-γ, two known inducers of sHLA-E as well as PMA and LPS which drive production of various cytokines like IL-1β, IL-6 and TNF-α from different cell types. Although all these agents induced sHLA-E shedding from ECV cells, the presence of sHLA-E in stimulated HFF culture supernatants (Fig. 4A) was not observed. These results also supported the possibility that the response of HFF to JEV and these stimulating agents was inherently different from the other cells used in the study.

Rabies, an RNA virus [38] has been shown to alter the expression of nonclassical HLA class I antigens such as HLA-E as well as HLA-G. Hence we tested the effect of poly (I:C) on sHLA-E shedding from ECV cells. The shedding of sHLA-E from ECV increased upon poly (I:C) treatment in a dose-dependent manner (Fig. 4B, top panel). Further, the activity of gelatinase also increased over time after poly (I:C) treatment which was only slightly lower than the increase obtained with a positive control such as TNF-α (Fig. 4B, middle panel). Since flaviviruses and poly (I:C) are reported to induce IFN-β [26], [39], we checked if IFN-β could also cause shedding of sHLA-E. As shown, treatment of ECV cells with IFN-β led to sHLA-E shedding that was inhibited with GM6001 (Fig. 4B, bottom panel). The comparative effects of JEV infection and poly (I:C) treatment of ECV cells are shown in Fig. S3. Although weaker than JEV in its ability to induce the IFN**-**β gene, poly (I:C) and JEV were similar in their ability to induce the TNF-α, IL-6 and MMP-9 genes. Poly (I:C) as well as TNF-α have also been reported to compromise the BBB in mice [40], and TNF-α as well as MMP-9 have been reported to be involved in sHLA-E release [32].

### Cytokines Induced by JEV Activate Release of sHLA-E

**Figure 5:**
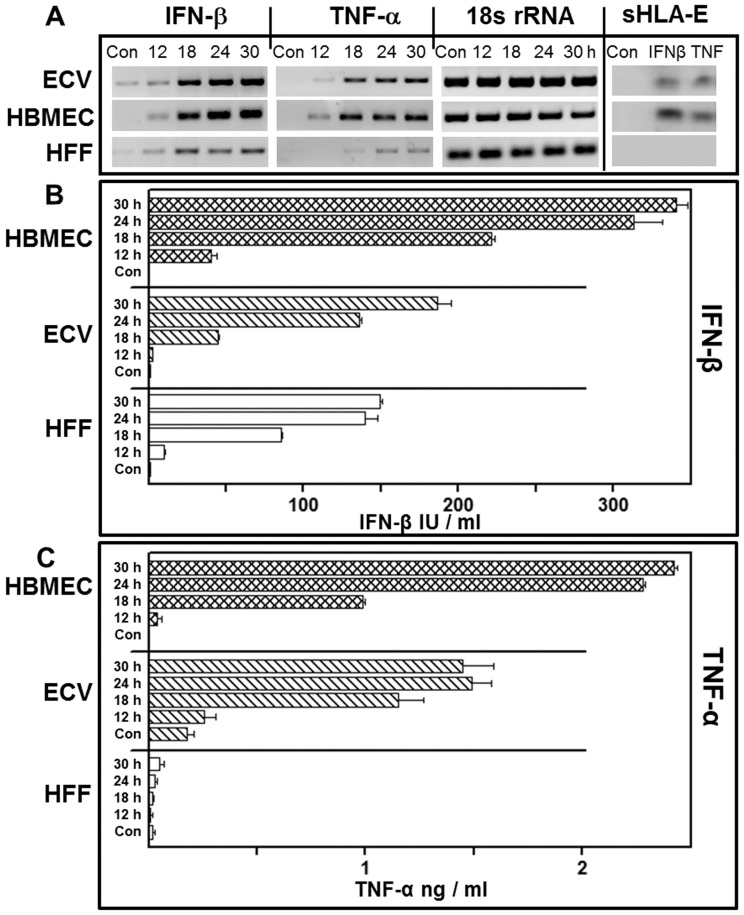
Analysis of IFN-β and TNF-α by RT-PCR and ELISA. Panel A: Total RNA was isolated from uninfected (Con) or JEV-infected ECV, HBMEC and HFF cells at the indicated times p.i. and subjected to semi-quantitative RT-PCR for IFN-β, TNF-α and 18 s rRNA as labeled. Last panel represents Western blotting for sHLA-E from cells that were untreated (Con) or treated with 500 IU of IFN-β and TNF-α for 24 h. Panel B and C: As indicated, bars represent the levels of IFN-β and TNF-α respectively in the supernatants of uninfected (Con), JEV-infected HBMEC (crossed bar), ECV (hatched bar) and HFF (open bar) at 12, 18, 24 and 30 h after infection. Values for uninfected HBMEC in both panels are negligible. Data represent mean levels of cytokine/ml ± SEM of triplicates.

MMP expression and sHLA shedding can be differentially activated by IFN-γ, TNF-α, IL-1β or other cytokines either when added alone or in combinations [33], [41]. We found that JEV infection of ECV cells could induce production of TNF-α and IFN-β but not IL-1β or IFN-γ (unpublished data). So, we analysed the expression of IFN-β and TNF-α by semiquantitative RT-PCR as well as ELISA after JEV infection. As shown in Fig. 5A, both IFN-β and TNF-α genes were induced progressively at different times after infection in ECV and HBMEC cells. HFF cells were also evaluated since they did not support productive JEV infection (Fig. 1 and Table. S1). Although the IFN-β gene was induced in infected HFF, the induction of the TNF-α gene was relatively low (Fig. 5A).

A time-dependent increase in the secretion of IFN-β (Fig. 5B) and TNF-α (Fig. 5C) from infected HBMEC (Fig. 5, crossed bars) and ECV cells (Fig. 5, hatched bars) was also observed. As expected from RT-PCR analysis (Fig. 5A), the secretion of TNF-α from HFF was negligible (Fig. 5C, open bars), while 150 IU/ml of IFN-β was secreted at 30 h after infection (Fig. 5B, open bars). In addition, treatment with IFN-β or TNF-α resulted in the shedding of sHLA-E as shown in Fig. 5A (last panel) suggesting that these cytokines induced the release of sHLA-E from ECV and HBMEC cells. Neither cytokine showed the presence of sHLA-E in the case of HFF cells.

### Maximum Inhibition of HLA-E and MMP-9 Gene Expression in JEV-infected ECV Requires both TNF-α and IFN-β

**Figure 6:**
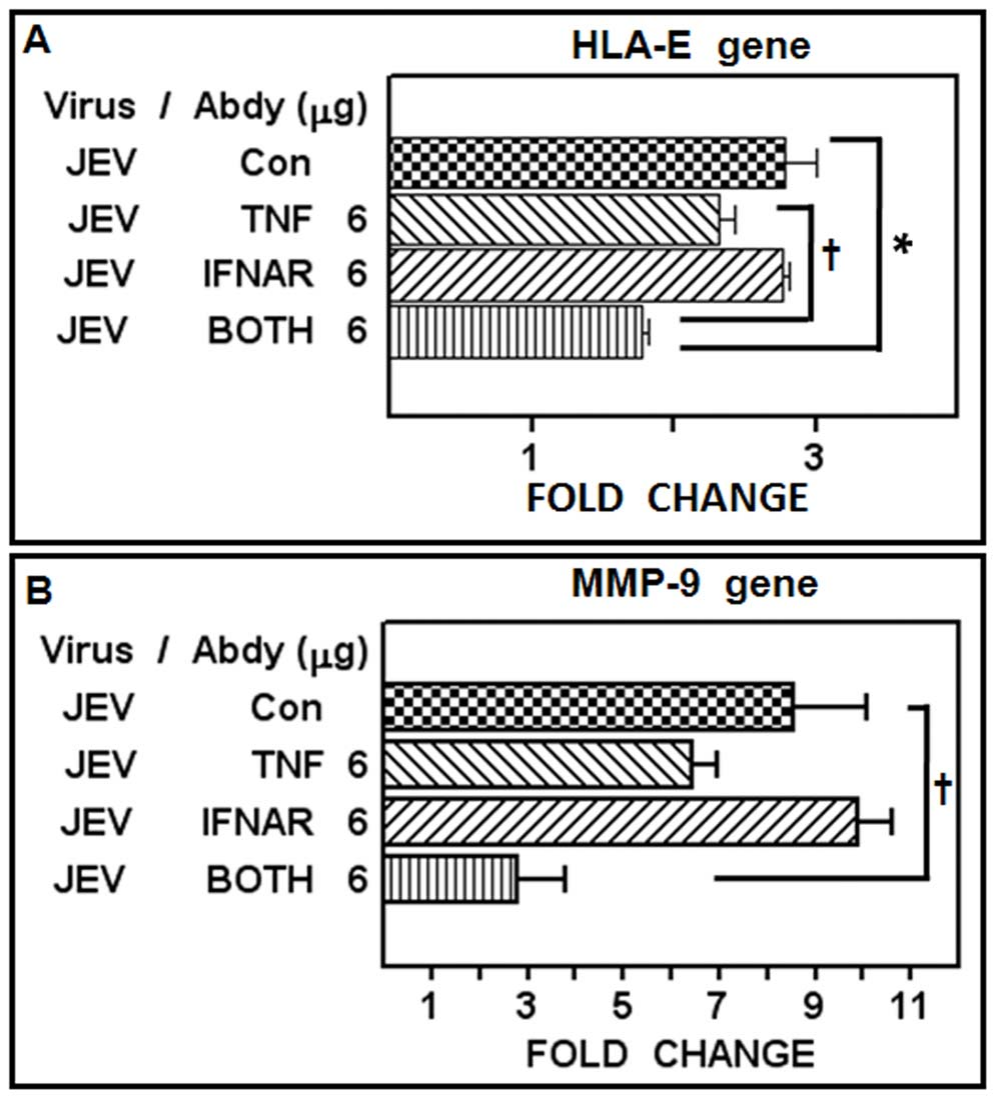
Inhibition of HLA-E and MMP-9 gene expression in JEV infected cells by anti-TNFα and anti-IFNAR antibodies. ECV cells were infected with JEV for 18 µg of anti-TNFα, anti-IFNAR antibodies or both as labeled and real time RNA quantification for HLA-E (Panel A) and MMP-9 (Panel B) genes was carried out. Controls (JEV Con) included infected cells cultured in the presence of 6 µg of the appropriate mouse isotype antibody. Data represent mean fold change over control RNA ± SEM (*P<0.001, ^†^P<0.05).

Both TNF-α and IFN-β were secreted upon JEV infection and both these cytokines also had the ability to induce shedding of sHLA-E from ECV cells. Hence we wished to test the effect of TNF-α and IFN-β inhibition on sHLA-E shedding from JEV infected cells. However, addition of anti-human cytokine antibodies into the culture made it difficult to visualize the presence of sHLA-E using anti-human secondary antibody reagent in Western blots. Therefore, the induction of HLA-E (Fig. 6A) and MMP-9 (Fig. 6B) genes were measured instead, by real-time analysis in ECV cells that were infected in the presence or absence of saturating antibodies to TNF-α and IFNAR. While the addition of 6 µg of anti-TNF-α neutralizing antibody alone inhibited the JEV-mediated induction of HLA-E and MMP-9 genes modestly, simultaneous addition of 6 µg each of anti-TNF-α and IFNAR antibodies significantly inhibited JEV induced upregulation of HLA-E and MMP-9 transcripts. Antibody mediated neutralization of TNF-α and IFNAR alone did not inhibit gene induction possibly because both TNF-α and IFN-β are secreted from infected cells (Fig. 5B and 5C). The requirement for both these cytokines in MMP-9 gene expression in JEV infected cells is presently unclear but these data suggest that both TNF-α and IFN-β may play a regulatory role in JEV-mediated HLA-E and MMP-9 gene induction.

### JEV-induced TNF-α and IFN-β Activate Release of sHLA-E from Uninfected Cells

**Figure 7:**
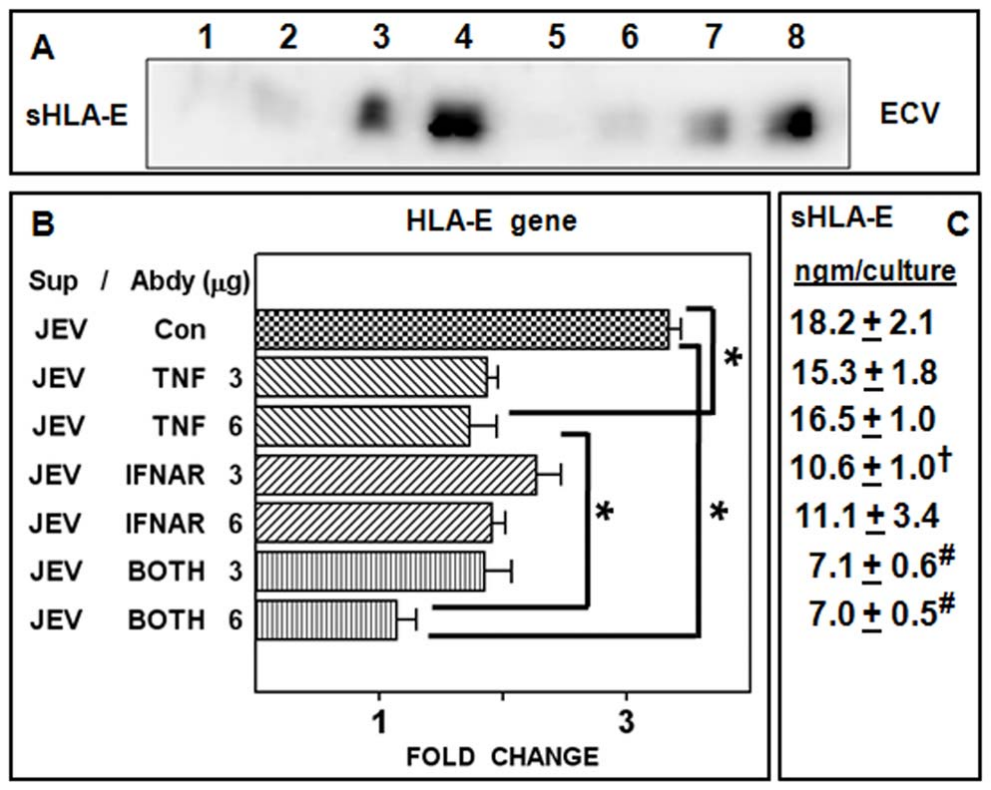
Stimulation of sHLA-E shedding and HLA-E gene expression in uninfected cells. Panel A: Uninfected (Lanes 3 and 7) and JEV infected ECV cells (Lanes 4 and 8) were cultured in the presence of untreated (Lanes 1 to 4) or UV inactivated culture supernatants (Lanes 5 to 8) obtained from 24 h-infected ECV cells. The supernatants harvested from these cultures after a period of 24 h were concentrated 10× and analysed for the release of sHLA-E by Western blotting on the same gel. Uninfected ECV culture supernatants were loaded on lanes 1 and 5 while the infected ECV culture supernatants used for stimulation were loaded on lanes 2 and 6. Panel B: UV inactivated culture supernatants obtained previously from 24 h-infected ECV cells were added to uninfected ECV cells and cultured in the presence of 3 µg and 6 µg of anti-TNFα, anti-IFNAR antibodies or both for 12 h. Cells were harvested and used for RNA extraction and analysis of HLA-E gene expression by real time quantification. Controls (JEV Con) included infected cells cultured in the presence of 6 µg of the appropriate mouse isotype antibody. Data represent mean fold change over control RNA ± SEM. Panel C: Cell culture supernatants were harvested and concentrated 10× and analysed for sHLA-E by ELISA. Data represent the mean sHLA-E released per culture ± SEM (*P<0.001, ^#^P<0.01, ^†^P<0.05).

We then asked, if TNF-α and IFN-β cytokines that were secreted upon JEV infection had the ability to induce HLA-E gene expression and activate sHLA-E release from uninfected cells. JEV infected cell culture supernatants were added to uninfected ECV cell cultures and sHLA-E release was monitored in the same Western analysis to obtain a better quantitative comparison. As shown in Fig. 7A, the addition of JEV-infected cell culture supernatants on uninfected ECV cells led to release of sHLA-E over and above the basal sHLA-E present in the added supernatant (Fig. 7A, Lanes 3 and 2). As expected, JEV-infected culture supernatants stimulated greater shedding of sHLA-E from infected ECV cells relative to uninfected cells (Fig. 7A, Lanes 4 and 3). Similar results were obtained when UV-inactivated cell culture supernatants were used (Fig. 7A, Lanes 5–8) suggesting that the increased release of sHLA-E from uninfected cells was not due to fresh infection by carry-over virus present in the added culture supernatants. Further, the addition of these supernatants did not result in sHLA-E release from uninfected or infected HFF cells (unpublished data) confirming earlier data (Fig. 4) that presence of sHLA-E could not be observed in infected HFF cell supernatants.

Our results with anti-TNFα and anti-IFNAR antibodies (Fig. 6) prompted us to examine the effect of neutralizing these two cytokines on HLA-E gene induction and sHLA-E shedding by real-time PCR (Fig. 7B) and ELISA (Fig. 7C) respectively. Uninfected ECV cells were stimulated with UV inactivated culture supernatants that were previously obtained from infected ECV cells. As shown in Fig. 7B, the addition of either anti-TNFα or anti-IFNAR antibodies to uninfected ECV cells that were treated with UV inactivated infected culture supernatants resulted in partial but significant inhibition of HLA-E gene induction. This partial inhibition was further potentiated when both antibodies were simultaneously present during the treatment. As shown in Fig. 7C, significant inhibition of sHLA-E shedding was obtained only when both anti-TNFα and anti-IFNAR antibodies were present in the culture suggesting that sHLA-E release was due to the combined effect of both TNF-α and IFN-β present in the infected culture supernatants. The lower sensitivity of this sHLA-E capture ELISA kit (∼6 ng per culture) made it difficult to obtain meaningful comparisons with earlier experiments involving JEV infected cells.

### Simultaneous Addition of Purified TNF-α and IFN-β Potentiates the Release of sHLA-E from Uninfected Cells

**Figure 8:**
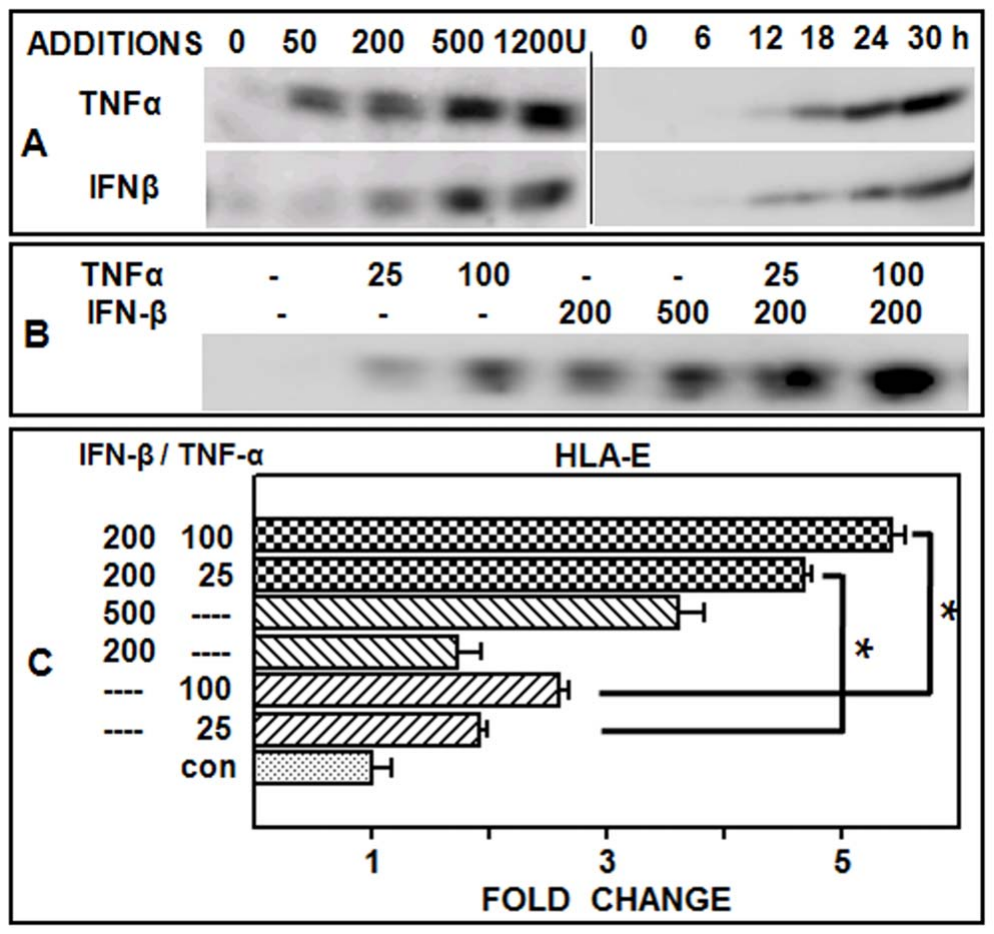
Enhanced release of sHLA-E from ECV by TNF-α and IFN-β. Panel A represents sHLA-E shedding from cells that were treated with either TNF-α or IFN-β and analysed by Western blotting using anti-HLA-E antibody. Treatment with 0, 50, 200, 500 and 1200 IU of cytokine for 24 h is shown on the left while results obtained with 500 IU at 0, 6, 12, 18, 24 and 24 h of treatment is on the right. Panel B represents sHLA-E shedding from cells treated for 30 h with TNF-α (25 and 100 IU) and IFN-β (200 and 500 IU), either alone or in combination. Panel C represents real time RNA quantification for the HLA-E gene. Cells were treated for 24 h with TNF-α (25 and 100 IU) and IFN-β (200 and 500 IU), either alone or in combination. Data represent mean fold change over untreated control (con) RNA ± SEM.

Although reported in melanoma patients [42], the ability of type I-IFNs to induce the shedding of sHLA-E has not been shown for endothelial cells. Moreover, optimal inhibition of the infected culture supernatant-stimulated sHLA-E release (Fig. 7), and JEV-induced HLA-E gene expression (Fig. 6) required the simultaneous addition of anti-TNF-α and anti IFNAR antibodies. Hence, we tested the effect of these two purified cytokines upon the shedding of sHLA-E from ECV cells when added alone or in combination (Fig. 8). While the treatment of ECV cells with either IFN-β or TNF-α alone increased sHLA-E shedding in a time and dose-dependent manner (Fig. 8A), combined addition of both cytokines resulted in an additive increase in sHLA-E shedding (Fig. 8B). The effect of IFN-β and TNF-α was then tested on the expression of the HLA-E gene (Fig. 8C). HLA-E gene expression increased with increased doses of either IFN-β or TNF-α, and was potentiated further in the combined presence of both cytokines. These data suggested that the two cytokines could potentiate sHLA-E shedding when present together. This also provided a plausible explanation for the requirement of both anti-TNFα and IFNAR antibodies for optimal inhibition of sHLA-E release from infected (Fig. 6) and uninfected ECV cells (Fig. 7).

## Discussion

In this study we demonstrate that, JEV infection in HBMEC and ECV cells resulted in production of IFN-β, TNF-α and MMP-9 with upregulation of MHC-I genes. These events required viral replication, as UV-inactivated virus failed to cause similar changes (Fig. S1A). Further, induction of HLA gene expression was followed by increased shedding of these molecules in HBMEC and ECV cells instead of increase in cell surface expression. On the contrary, JEV induced cell surface alterations of HLA-E were evident in infected HFF cells (Fig. 2), which do not release soluble HLA E, suggesting a different mechanism that operate in these cells upon JEV infection. The ability to stimulate cell surface HLA-E and IFN-β secretion but not TNF-α (Fig. 2B and 5) despite an inability to support a productive virus life cycle (Table. S1) also suggested that infected HFF cells were different. Such abortive infections with nonproductive virus life cycles have been shown in other viruses to alter cytokine secretion and influence biological consequences at the local sites of infection [29], [43].

The upregulation of MHC-I by JEV is dependent solely on type 1-IFNs in mouse embryonic fibroblasts [30] while it is dependent on both type 1-IFNs and NF-κB in the case of WNV [44]. The regulation of HLA-E is known to be independent of NF-κB [45] but is inducible by TNF-α [42], a cytokine known to be upregulated during JEV infections [46] and confirmed here. Both IFN-β and TNF-α were not only produced from infected endothelial cells but they were also able to induce the HLA-E gene in uninfected cells. Hence we examined the effect of these cytokines on the shedding response further. TNF-α and IFN-β were found to induce a robust shedding response in ECV cells which was more pronounced in the presence of both cytokines (Fig. 8). In addition, this potentiating effect was also observed with HLA-E gene induction. Taken together with the cytokine neutralization experiments, these data suggest that both IFN-β and TNF-α play a role in sHLA-E shedding.

Our data with MMP inhibitors showed that GM6001 inhibited JEV-induced shedding of sHLA-E from infected ECV and HBMECs. In addition, the sHLA-E released by TNF-α and IFN-β was inhibited by GM6001 (Fig. 3C and 4B). LPS and PMA, agents that activate MMP-9 secretion [41] also caused sHLA-E shedding in our study (Fig. 4A and Fig. S3). Low levels of constitutive MMP-9 activity, either released from ECV cells or present in media supernatants could also be responsible for sHLA-E shedding when MMP-9 gene induction may be absent. Low levels of constitutive MMP-9 gene expression were also observed in normal ECV cells (Fig. S3). MMP-9 has also been reported to be upregulated in JEV infected brain astrocytes [37], [47] and the shedding of sHLA-E from endothelial cells that is activated by TNF-α, IL-1 and IFN-γ in vascular diseases is MMP-9 dependent [32]. Hence our observation that MMP-9 gene expression (Table. 2) and gelatinase activity (Fig. 3A) was upregulated upon JEV infection as well as the inhibition obtained with GM6001 suggests that MMP-9 may contribute, at least partially to the release of sHLA-E from JEV-infected endothelial cells. The heavy chains of HLA class I molecules are also bound to plasma lipoproteins, secreted as alternatively spliced forms or shed as soluble forms including exosomes and those that are produced due to proteolytic cleavage by MMPs [34], [48], [49]. While previous reports supporting a role for MMPs in the shedding of HLA-E from melanoma cells encouraged us to focus on a possible role for MMPs in this study, the participation of other mechanisms and JEV proteins in this shedding process has not been investigated.

Cytokines such as TNF-α and IL-6 that were induced in our study (Fig. 5 and Fig. S3) are also known to alter the permeability of endothelial cells [26], [50]. JEV infection of rat brain microvascular endothelial cells has recently been shown to make them favorable to leukocyte adhesion [10]. Our studies also show that TNF-α and IFN-β secreted in response to JEV infection have the ability to act on neighboring uninfected cells in a paracrine manner leading to HLA-E gene induction and release of sHLA-E. The partial inhibition obtained with anti-TNFα and IFNAR antibodies suggests that other cytokines may also contribute to sHLA-E release. Hence these effects of JEV shown in our study with HBMEC and ECV cells finally leading to TNF-α and MMP-9 release may be one of the indirect causes of damage and leukocyte infiltration across infected and uninfected vicinal endothelial cells. GM6001 has been shown to reverse the WNV-induced degradation of human brain endothelial tight junction proteins [12]. In fact, TNF-α and IL-6 have been shown to contribute to the leakiness of the BBB caused by the TLR-3 mediated infection of mice with WNV [40].

Further study is needed to identify the exact role of sHLA-E and other sHLA molecules during JEV infection. However, the findings that sHLA-E inhibited lysis by NK cells [33] and that other sHLA molecules cause apoptosis of CD8^+^ NK and T cells [51] seem to suggest an immune evasive role for this JEV-induced shedding response.

## Materials and Methods

### Media, Antibodies, and Cell Lines

Media and reagents were purchased from Sigma-Aldrich India. Gelatin was from Merck, India. FCS was obtained from Gibco, USA and Nu-Serum from BD Biosciences, USA. Revert Aid Moloney murine leukemia virus (M-MuLV) reverse transcriptase and Taq polymerase were obtained from MBI Fermentas, Canada and iQ SYBR Green Supermix 2X was purchased from Bio-Rad, USA. Anti HLA-E antibodies MEM-E/07 and MEM-E/02 were obtained from Abcam, USA. W6/32, a pan HLA class I-specific FITC conjugated monoclonal antibody that detects only conformed HLA was obtained from Exbio, Czech Republic while goat anti-mouse IgG (Fcγ chain specific) secondary antibody conjugated to FITC was from Jackson Immunoresearch Labs, USA. Anti JEV-NS3 antisera was raised by the immunisation of rabbits with overexpressed and purified NS3 protein. Goat anti human β-tubulin for Western blotting and donkey anti goat IgG-HRP conjugate were obtained from Santacruz Biotech, USA. Goat anti mouse IgG-HRP and goat anti rabbit-HRP were from Bangalore Genei, India. Galardin (GM6001), a broad spectrum MMP inhibitor, was obtained from Merck, USA while leupeptin and PMSF were obtained from Sigma-aldrich, India. ELISA kits for detecting TNF-α and IFN-β were obtained from Peprotech, Israel and Invitrogen, USA respectively. Human TNF-α, IFN-β and IFN-γ were from Peprotech, Israel. Rabbit polyclonal anti-human TNF-α antibody was obtained from Peprotech, Israel and mouse monoclonal anti human interferon alpha/beta receptor chain 2 antibody was obtained from PBL interferon source, USA.

Human Brain Microvascular Endothelial cells (HBMEC) transfected with a plasmid containing the SV40 Large T antigen [52] were obtained from Dr. Kim and cultured in DMEM containing 10% FCS, 10% Nu-Serum, glutamine, non-essential aminoacids, sodium pyruvate and MEM vitamin solution. ECV304 was a gift from Dr. M. Jaggi, Dabar Research Center, Ghaziabad (hereafter referred to as ECV). ECV is an endothelial-like cell line originally identified as a human umbilical endothelial cell [53] and later found to be a bladder carcinoma-derived epithelial cell [54]. HFF cell line was a kind gift for this study from Dr. Shobhana Sharma, DBS, TIFR, Mumbai [55]. The human amniotic epithelial cells, AV-3 and FL were obtained from NCCS, Pune, while WISH, was a gift from Dr. Ajit Kumar, MCB department, Indian Institute of Science, Bangalore [56]. They were grown in DMEM containing 10% FCS.

### Virus and Infection of Cells

JEV strain P20778, was propagated in C6/36 mosquito cells and virus titers were determined by plaquing on PS cells [30]. Virus infection was carried out by adsorbing cells with virus at MOI 10 for 2 h at 37°C. Virus was removed, fresh medium containing 2.5% FCS was added and considered as 0 h infection. Thereafter, the cells were harvested at different times post infection (p.i.) and used for FACS staining, cell extract preparation and RNA isolation. Cell-free culture supernatants were collected from infected cells at different times p.i. and stored at −70°C until tested. For soluble HLA, culture supernatants were harvested from 6 well plates containing 7.5×10^5^ cell/ml and concentrated 10× on 10 kDa cut off centricons (Millipore).

### Treatment of ECV with Inhibitors, Activating Agents and Cytokines

ECV cells (7.5×10^5^) were adsorbed with JEV in 6-well plates as given above, washed and cultured for 24 h in 1 ml fresh medium containing 2.5% FCS. The following inhibitors were added in fresh medium at 18 h of culture i.e., during the last 8 h of culture: GM6001 (5 and 10 µM), leupeptin (10 µM), PMSF (1 mM) or chloroquine (100 µM). Poly I:C (10, 50 and 100 µg), PMA (100 ng), LPS (100 µg), IFN-γ, IFN-β and TNF-α (500 IU/ml) were added for 24 h unless otherwise mentioned. DMSO and ethyl alcohol were used as solvent controls and did not differ from untreated controls.

### Stimulation of ECV Cells with JEV Infected Cell Culture Supernatants

For the purpose of stimulating normal ECV cell cultures, JEV infected culture supernatants were harvested from 6 well plates containing infected 7.5×10^5^ cells/ml after 24 h of infection. They were spun at 10,000×g and stored at −70°C before being UV inactivated for use in experiments. The presence of sHLA-E in these supernatants was confirmed by Western blotting after concentrating them 10×. One ml of this unconcentrated (1×) supernatant was added directly on normal ECV cells (7.5×10^5^ cells/ml) that were either cultured alone or infected with JEV (MOI 10) for a period of 18 h. Cell culture supernatants were collected after a further period of 12 h, clarified by spinning at 10, 000×g, concentrated 10× on 10 kDa cut off centricons and analysed by Western blotting for sHLA-E as described below.

In a separate experiment, uninfected ECV cells were also cultured in 12 well plates with 0.5 ml of unconcentrated (1×) UV inactivated JEV infected cell culture supernatants that were collected from 7.5×10^5^ cells/ml cultures as given above. Cells were harvested from such treated uninfected ECV cultures after 12 h and used for RNA extraction, cDNA preparation and real time PCR analysis. Cell culture supernatants were also collected and concentrated 10× for evaluation of sHLA-E release by ELISA. Saturating amounts of anti-TNFα, anti-IFNAR or control antibodies (6 µg or 3 µg) were added, either alone or in combination during the 12 h period. The appropriate mouse isotype antibody (6 µg) was used as the control. The addition of normal polyclonal antibody did not show any inhibitory effect on sHLA-E release (unpublished data) and hence were not used for all studies.

ECV cells (2×10^5^) when infected directly were adsorbed for 2 h with JEV (MOI 10) in 12-well plates containing 0.5 ml as given above, washed and further cultured for 16 h in 0.5 ml fresh medium containing 2.5% FCS in the presence or absence of antibodies. Saturating amounts (6 µg) of anti-TNFα, anti-IFNAR or control antibodies were added either alone or in combination and the cells were harvested for RNA extraction and real-time PCR analysis.

### Semiquantitative Reverse Transcriptase (RT-PCR) Analysis

Total RNA was isolated from infected and control cells using Trizol (Sigma-aldrich, India) reagent according to the manufacturer's instructions. First strand cDNA was made from 2 µg of RNA using Revert Aid™ M-MuLV Reverse Transcriptase from MBI Fermentas, Canada and oligo dT or random primers from MBI Fermentas, Canada. PCR was performed with 1∶25 diluted cDNA using gene-specific primers in a reaction volume of 50 µl using a Peltier thermal cycler from Bio-Rad, USA. PCR was performed for 24 cycles in the case of JEV Envelope and 18 s rRNA, 32 cycles in the case of HLA-E, IL-6, TNF-α and IFN-β while 40 cycles were performed for MMP-9. The gene specific primers and the number of cycles used for semi-quantitative PCR are listed in Table. S3. Prior standardization experiments were performed to ensure the semiquantitative nature of the results obtained which were confirmed in at least two different experiments.

### Quantitative Real Time PCR Analysis

The real-time PCR for the relative quantification of genes was performed using a Bio-Rad iQ5 system (Bio-Rad, USA). Real-time PCR mixtures contained 10 µl of 2× iQ SYBR Green Supermix (Bio-Rad, USA), 0.75 µl of each primers, 4 µl of 1∶50 diluted cDNA and water in a final volume of 20 µl. Relative fold change was determined using the threshold cycle (2^−ΔΔCt^) method where 18 s rRNA was used as a reference gene for normalization. The gene-specific primers and the thermal cycling conditions used are listed in Table. S4. Data was analysed by one-way ANOVA with Dunnett’s post test using GraphPad Prism version 5.00 and represented as mean fold change ± SEM of three independent PCR analyses.

### Western Blot

Cells (1×10^6^) were lysed in 90 µl lysis buffer (50 mM Tris-HCl, 1% NP-40, 0.25% Sodium Deoxycholate, 150 mM NaCl, 1 mM EDTA, 1 mM PMSF, 1 mM Aprotinin, 1 mM Leupeptin, 1 mM NaF) at 4°C for 30 min. The suspension was spun at 12,000×g for 15 min and 100 µg protein aliquots were electrophoresed on 10% native or SDS-PAGE gels and blotted onto Millipore Immobilon-P PVDF membranes.

For soluble HLA class I and soluble HLA-E (sHLA-E), equal aliquots of 10× concentrated media supernatants were used. Membranes were blocked and incubated with optimal titers of primary mouse anti HLA-E antibody, MEM/02 or anti-tubulin antibody. The blot was developed using Immobilon western chemiluminescent HRP substrate (Millipore, India) and visualized using a Luminescent Image Analyzer (LAS 3000, Fuji Film, Japan) after incubation with HRP conjugated secondary antibody. Blots shown in Fig. 1 and 2 used the same control since they were first probed for HLA-E, stripped and then reprobed for JEV-NS3 and tubulin as loading control. These data were confirmed in three independent experiments.

### ELISA

Cells (7.5×10^5^/ml) were infected at MOI 10 and culture supernatants that were collected at different times p.i. were cleared by spinning at 3000×g for 15 min and stored at -70°C until processed. ELISA was performed according to manufacturer’s instructions using Human TNF-α ELISA kit from Peprotech, Israel and Human Interferon-β ELISA kit from Fujirebio Inc, Japan. Human Leukocyte Antigen E (HLA-E) ELISA kit for the assay of HLA-E in cell culture supernatants was obtained from TSZ ELISA, Massachusetts, USA and used with minor modifications.

### Flow Cytometry

Viable cells (0.3×10^6^) were stained and analysed [30] using a FACSCalibur Cytometer (Becton Dickinson, USA). Data for 10,000 events was analysed using WinMDI software (Version 2.8). Cell surface expression of total HLA class I was analysed using the FITC conjugated mouse anti pan HLA class I-specific primary antibody, W6/32 and the appropriate FITC conjugated isotypic secondary antibody. Mouse anti HLA-E antibody MEM/07 clone and FITC conjugated goat anti mouse Fcγ antibody were used as primary and secondary antibodies to stain cell surface HLA-E. Control and virus infected cells were stained at different times p.i. and the time at which maximum changes were observed is plotted. Data is representative of four independent experiments.

### Zymography for Gelatinase Activity

ECV cells (7.5×10^5^/ml) were infected in 6 well plates at MOI 10 for 2 h at 37°C. Virus was removed and the cells were cultured in fresh medium containing 2.5% FCS. After 18 h, media was replaced with DMEM without serum. Thereafter, media supernatants were collected after 4 h (22 h p.i.) and 8 h (26 h p.i.) to measure gelatinase activity. Aliquots (40 µl) were mixed with non-reducing 6× SDS loading dye, incubated at room temperature for 30 min and electrophoresed on 8% SDS-PAGE gel containing 0.1 mg/ml gelatin. The gel was washed twice with 2.5% Triton X-100 at 37°C for 30 min each, incubated for 36 h at 37°C in incubation buffer (10 mM Tris-HCL pH 7.5, 2.5% Triton X-100, 5 mM CaCl_2_ and 1 µM ZnCl_2_) followed by staining with 0.04% Coomassie Brilliant Blue stain and destained until bands developed. Gelatinase activity in ECV cells was also measured at 26 h and 24 h after treatment in the case of IFN-γ and TNF-α respectively. In the case of poly (I:C), the activity was measured at 22 h and 26 h after treatment. Activity staining experiments were confirmed several times.

## Supporting Information

Figure S1 SDS PAGE analysis of total cellular and soluble HLA-E. Panel A: As labeled on the left, 100 µg of ECV, AV-3, FL and WISH total cell lysates was subjected to Western blotting analysis using anti HLA-E, MEM/02 (Top) to detect total cellular HLA-E (42 kDa) and anti β-tubulin antibody (Bottom) as control. Control uninfected cells (Con) and cells that were infected with either UV inactivated JEV (UV-JEV) or active JEV (JEV) at MOI 10 for 24 h are shown. Panel B: 100 µg of cell lysate protein (Lanes 1, 4, 6) and equal aliquots of cell culture supernatants (Lanes 2, 3, 5) were obtained from cells that were infected with JEV for 24 h at MOI 10 as described in Materials and Methods. They were separated on 12.5% SDS PAGE gels and subjected to Western blotting using anti-JEV NS3 antiserum (Lane 1) or anti-HLA-E, MEM/02 antibody that detects denatured HLA-E. Lanes 1, 2 and 5 represent ECV cells while lane 3 shows sHLA-E from HBMEC cells. Lanes 4 and 6 show total cell lysates prepared from ECV and FL cells respectively. Arrows represent the position of JEV NS3 protein (71 kDa), sHLA-E (37 kDa) and total cellular HLA-E (42 kDa) antigens.
(TIF)

Figure S2 Native PAGE analysis for sHLA class I shedding by JEV-infected cells. Equal aliquots of cell-culture supernatants from ECV (Lanes 1, 2, 5, 6, 9, 10) and HFF (Lanes 3, 4, 7, 8) cells were separated on 10% native PAGE gels and subjected to Western blotting for HLA-class I (Panel A, C) or HLA-E (Panel B, D). Panels A and B represent JEV infected cells where lanes 1, 3, 5, and 7 represent uninfected cells and lanes 2, 4, 6 and 8 represent JEV-infected cells. Panels C and D represent cells treated with 500 IU IFN-γ for 24 h as positive controls. Arrows show the position of sHLA class I and sHLA-E.
(TIF)

Figure S3 Quantification of gene expression in ECV by RT-PCR analysis. As labeled, total RNA was isolated from control (Con) and 24 h JEV-infected as well as 24 h after treatment with LPS (100 µg), p(I:C)-100 µg and PMA (100 ng). Semi-quantitative RT-PCR was performed using gene specific primers and electrophoresed on 2% agarose gels.
(TIF)

Table S1 JEV virus titers in infected cells.
(TIF)

Table S2 Virus titers after treatment with inhibitors.
(TIF)

Table S3 Semiquantitative RT-PCR analysis.
(TIF)

Table S4 Quantitative Real Time RT-PCR analysis.
(TIF)
