# Supplementary figures and images for: Infection of Human Endothelial Cells by Japanese Encephalitis Virus: Increased Expression and Release of Soluble HLA-E
Source: PLoS One. 2013 Nov 13;8(11):e79197. doi: 10.1371/journal.pone.0079197 (PMC3827286; doi:10.1371/journal.pone.0079197)

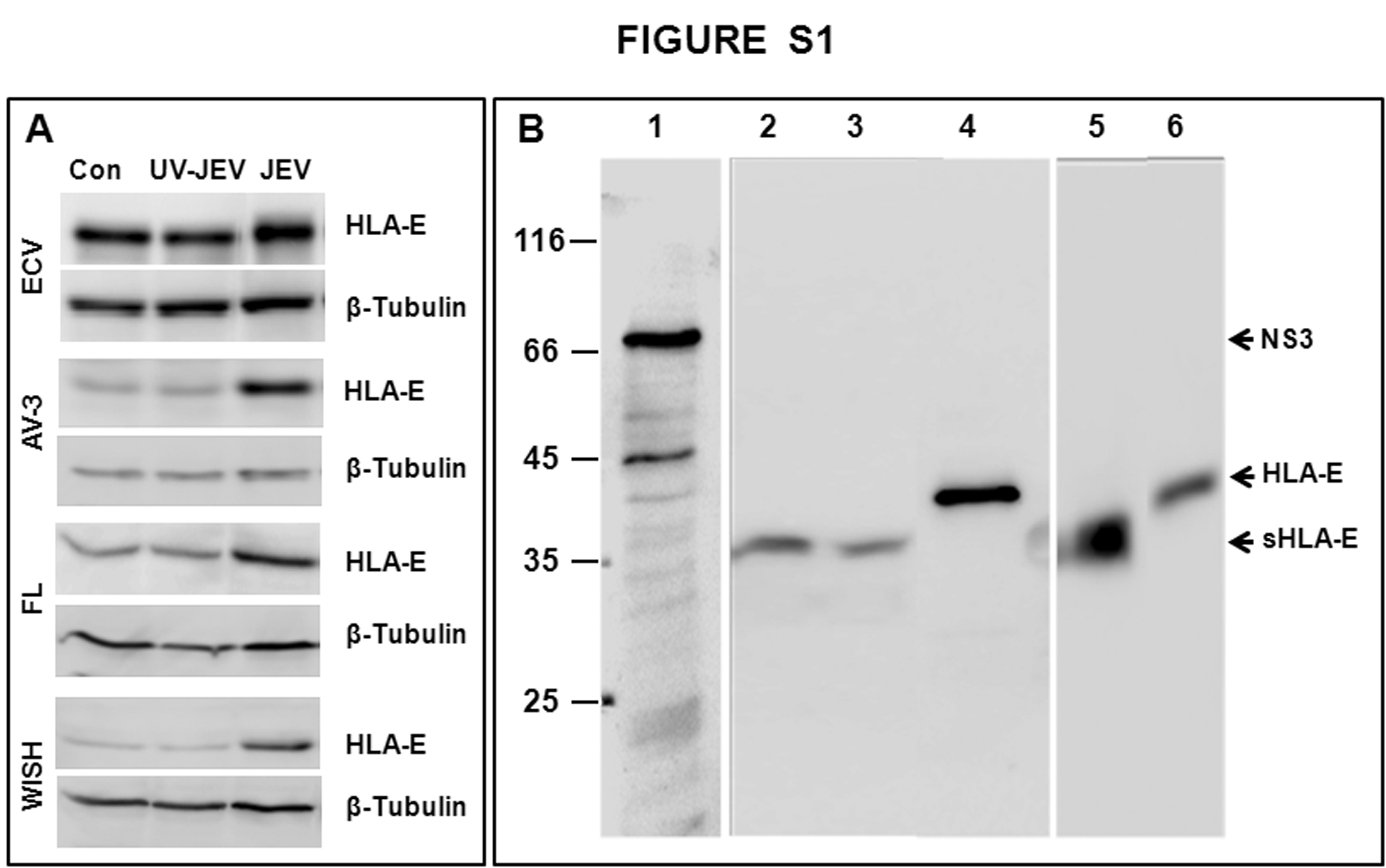

Supplement: Figure S1 — SDS PAGE analysis of total cellular and soluble HLA-E. Panel A: As labeled on the left, 100 µg of ECV, AV-3, FL and WISH total cell lysates was subjected to Western blotting analysis using anti HLA-E, MEM/02 (Top) to detect total cellular HLA-E (42 kDa) and anti β-tubulin antibody (Bottom) as control. Control uninfected cells (Con) and cells that were infected with either UV inactivated JEV (UV-JEV) or active JEV (JEV) at MOI 10 for 24 h are shown. Panel B: 100 µg of cell lysate protein (Lanes 1, 4, 6) and equal aliquots of cell culture supernatants (Lanes 2, 3, 5) were obtained from cells that were infected with JEV for 24 h at MOI 10 as described in Materials and Methods. They were separated on 12.5% SDS PAGE gels and subjected to Western blotting using anti-JEV NS3 antiserum (Lane 1) or anti-HLA-E, MEM/02 antibody that detects denatured HLA-E. Lanes 1, 2 and 5 represent ECV cells while lane 3 shows sHLA-E from HBMEC cells. Lanes 4 and 6 show total cell lysates prepared from ECV and FL cells respectively. Arrows represent the position of JEV NS3 protein (71 kDa), sHLA-E (37 kDa) and total cellular HLA-E (42 kDa) antigens. (TIF) [file pone.0079197.s001.tif]

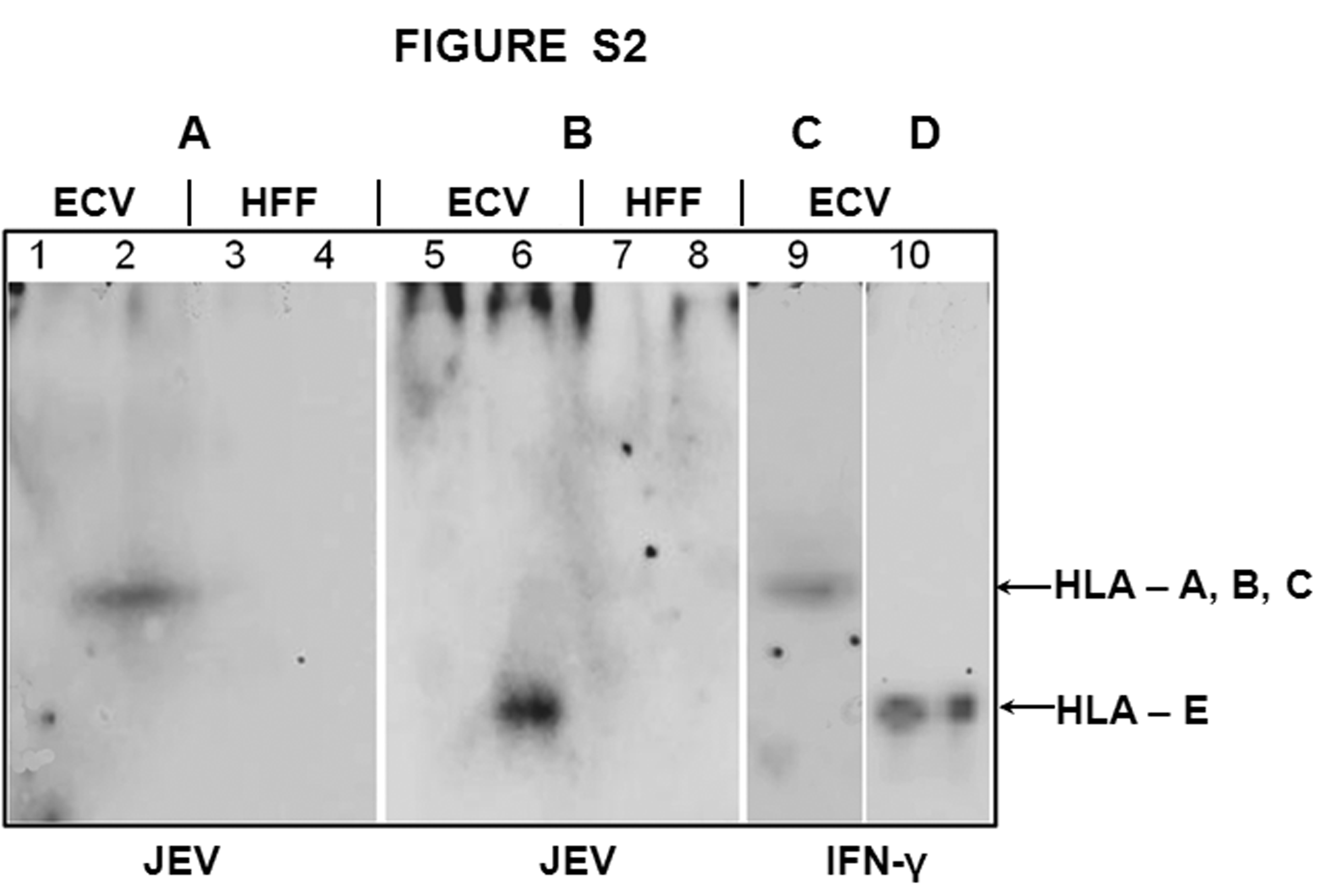

Supplement: Figure S2 — Native PAGE analysis for sHLA class I shedding by JEV-infected cells. Equal aliquots of cell-culture supernatants from ECV (Lanes 1, 2, 5, 6, 9, 10) and HFF (Lanes 3, 4, 7, 8) cells were separated on 10% native PAGE gels and subjected to Western blotting for HLA-class I (Panel A, C) or HLA-E (Panel B, D). Panels A and B represent JEV infected cells where lanes 1, 3, 5, and 7 represent uninfected cells and lanes 2, 4, 6 and 8 represent JEV-infected cells. Panels C and D represent cells treated with 500 IU IFN-γ for 24 h as positive controls. Arrows show the position of sHLA class I and sHLA-E. (TIF) [file pone.0079197.s002.tif]

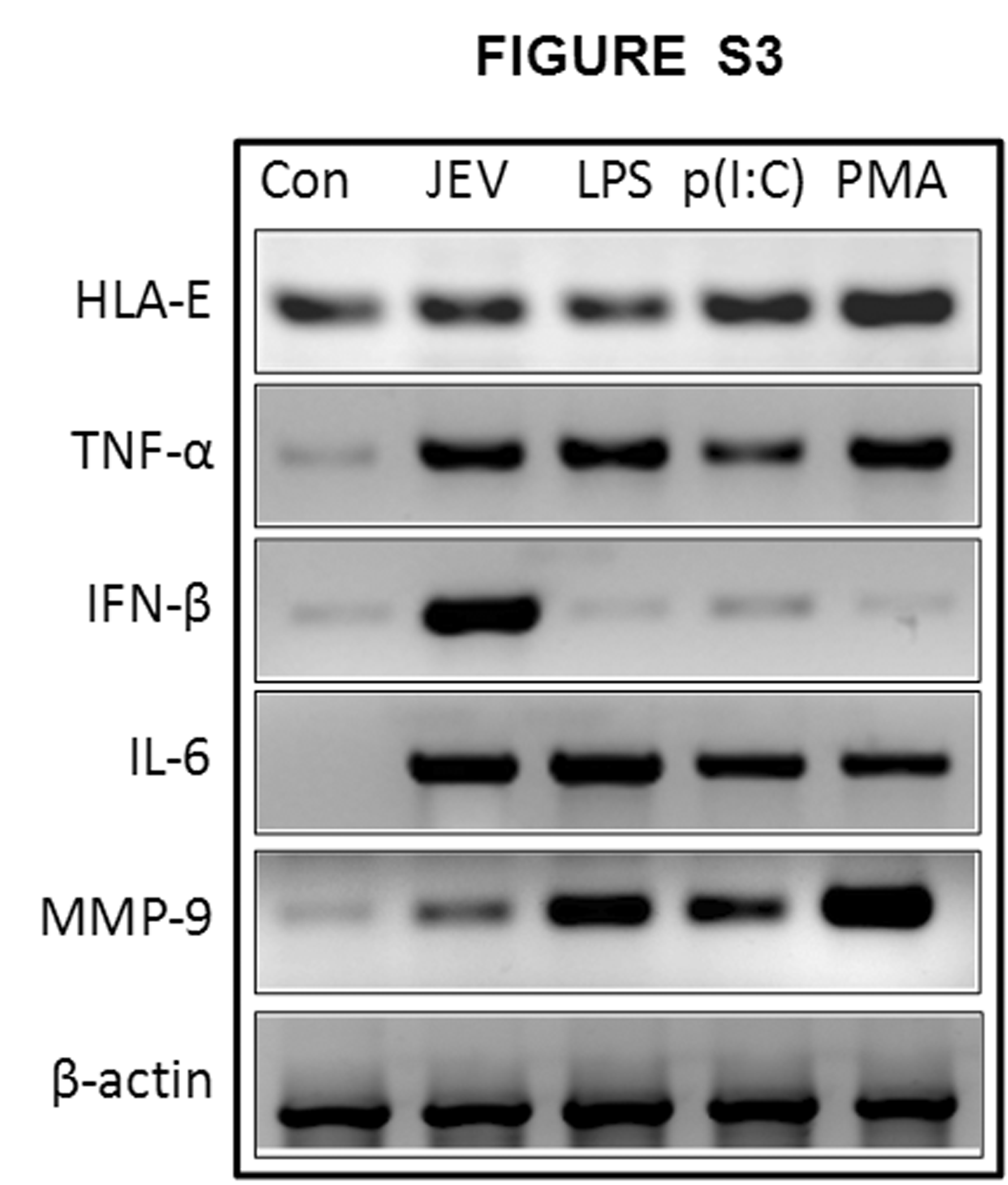

Supplement: Figure S3 — Quantification of gene expression in ECV by RT-PCR analysis. As labeled, total RNA was isolated from control (Con) and 24 h JEV-infected as well as 24 h after treatment with LPS (100 µg), p(I:C)-100 µg and PMA (100 ng). Semi-quantitative RT-PCR was performed using gene specific primers and electrophoresed on 2% agarose gels. (TIF) [file pone.0079197.s003.tif]

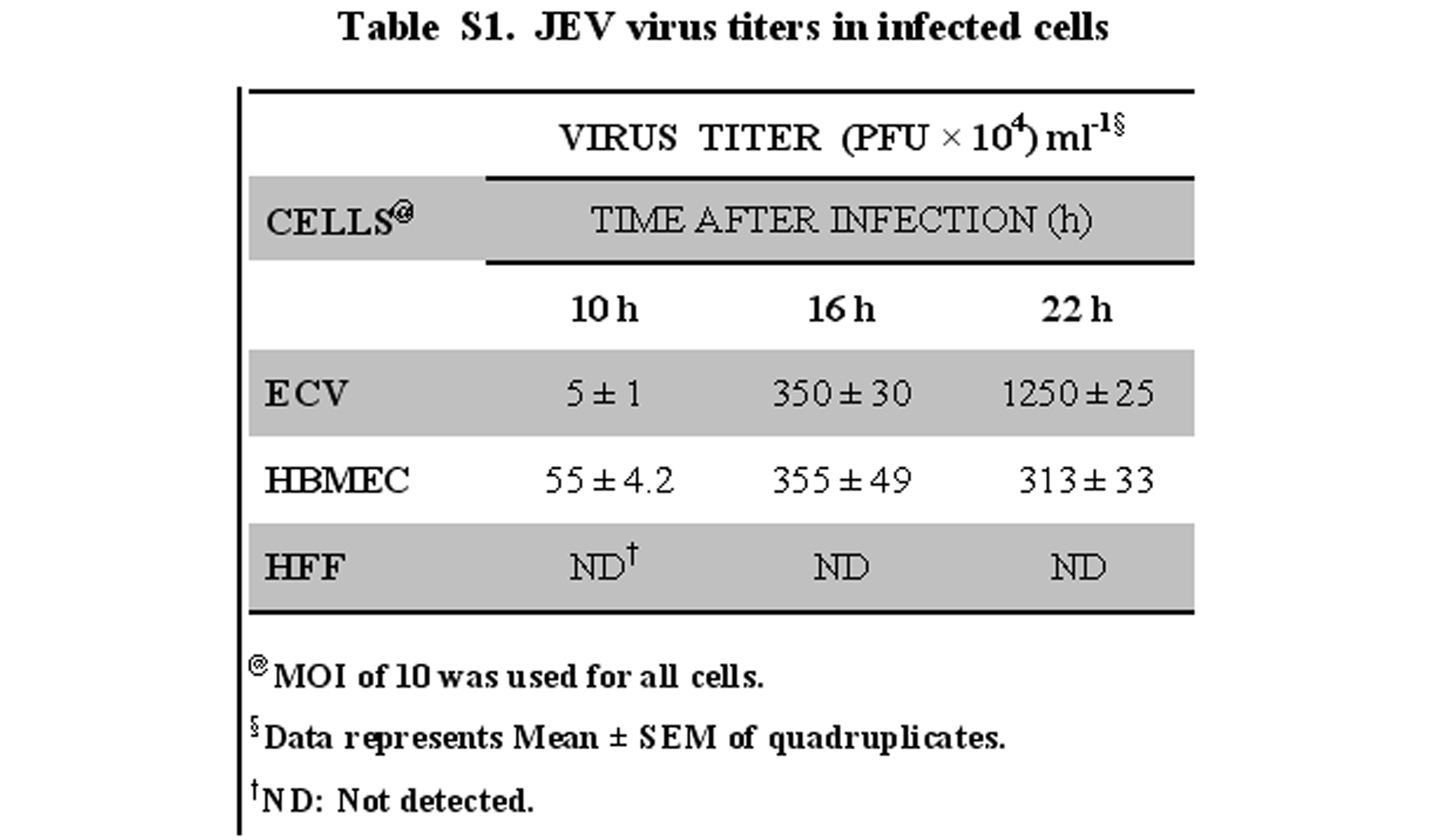

Supplement: Table S1 — JEV virus titers in infected cells. (TIF) [file pone.0079197.s004.tif]

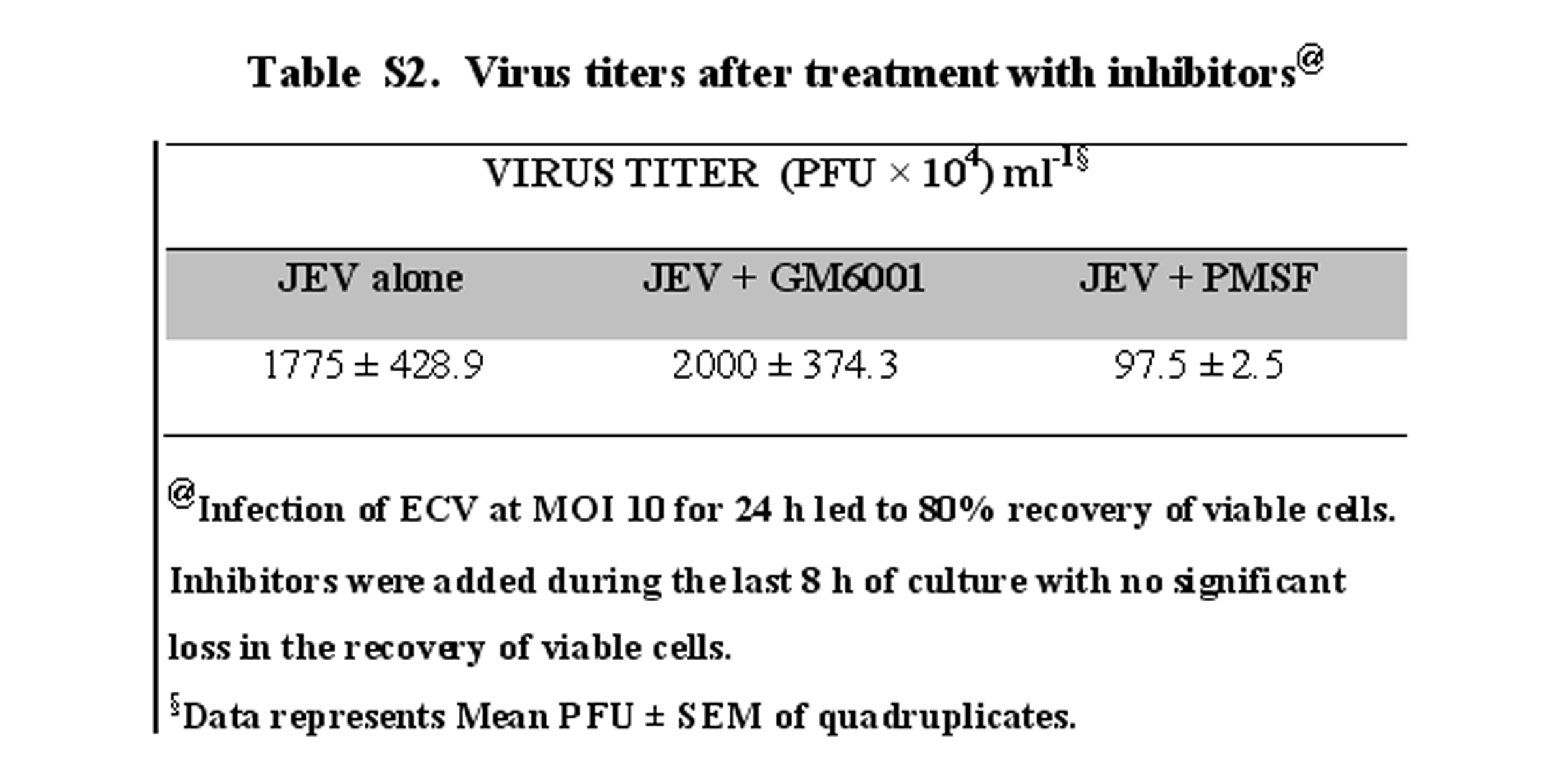

Supplement: Table S2 — Virus titers after treatment with inhibitors. (TIF) [file pone.0079197.s005.tif]

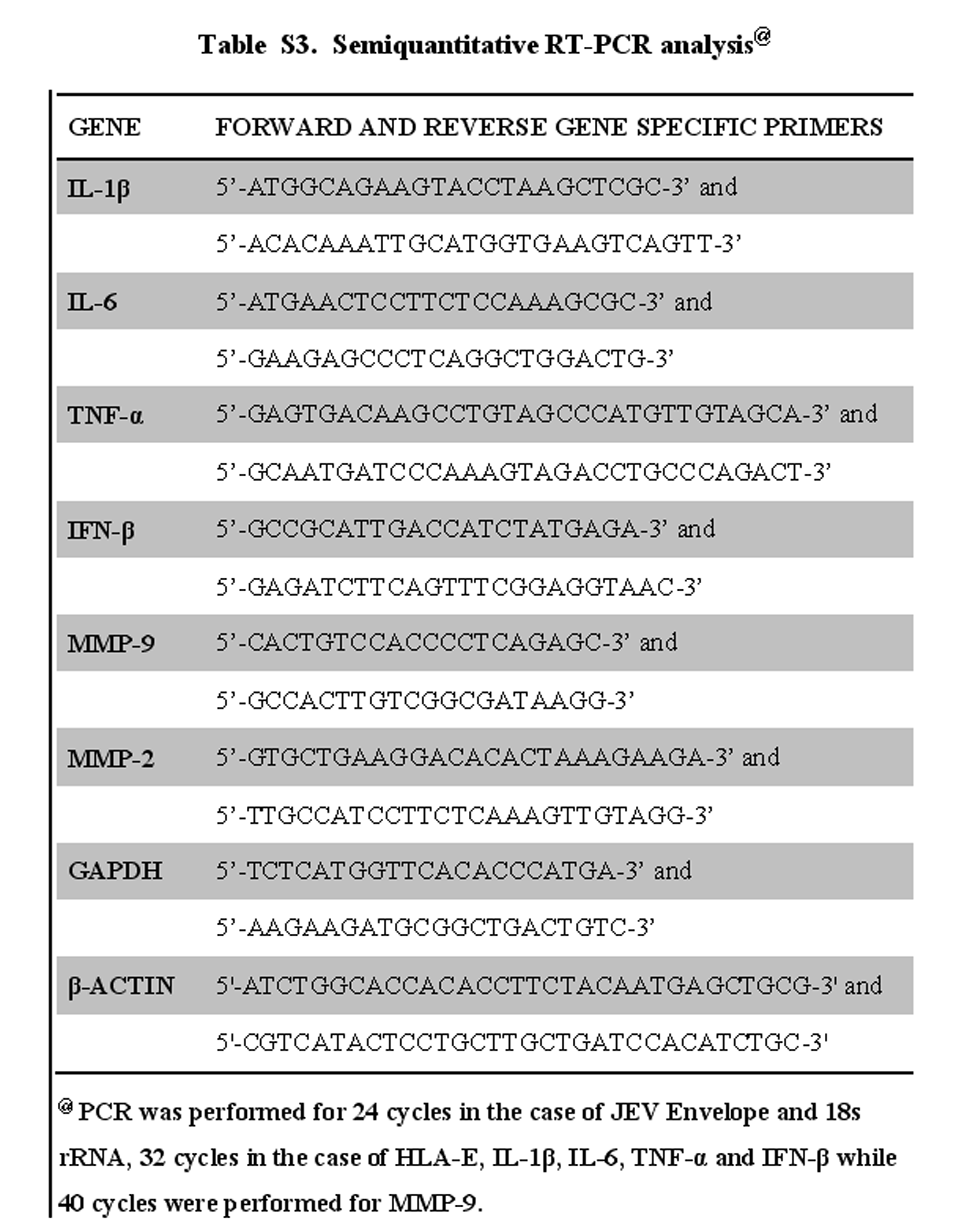

Supplement: Table S3 — Semiquantitative RT-PCR analysis. (TIF) [file pone.0079197.s006.tif]

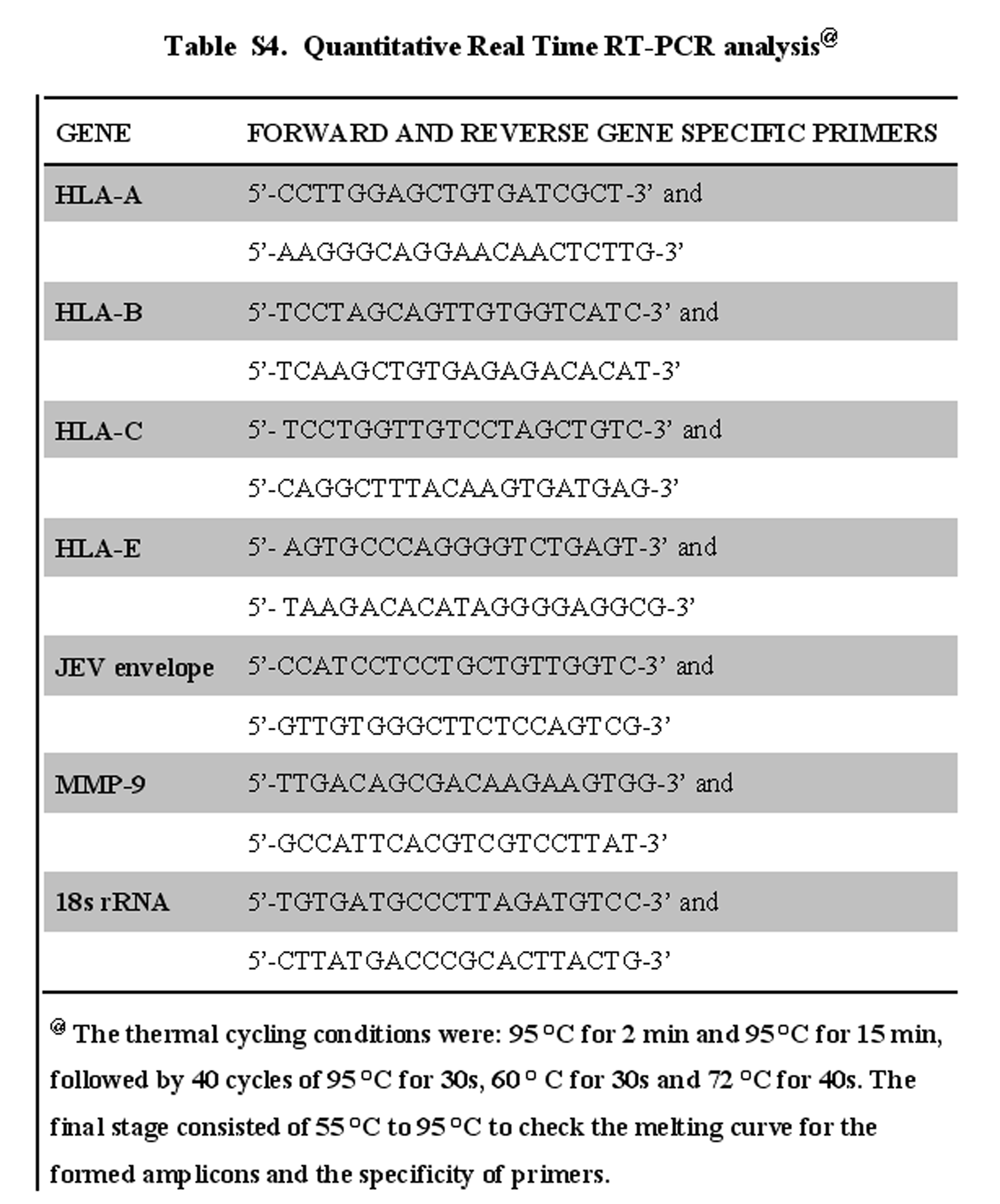

Supplement: Table S4 — Quantitative Real Time RT-PCR analysis. (TIF) [file pone.0079197.s007.tif]
